# ARIPIPRAZOLE INDUCED COMPULSIVE EATING DISORDER: A CASE REPORT

**DOI:** 10.1192/j.eurpsy.2023.1795

**Published:** 2023-07-19

**Authors:** M. Turki, A. Samet, F. Sahnoun, F. Jemil, S. Ellouze, N. Halouani, J. Aloulou

**Affiliations:** Psychiatry « B », Hedi Chaker university hospital, sfax, Tunisia

## Abstract

**Introduction:**

Aripiprazole is a newer atypical antipsychotic with a favorable side-effect profile, especially a low propensity to result in metabolic syndromes.It is effective at treating bipolar disorder, the positive symptoms of schizophrenia and has the potential to treat negative and cognitive symptoms.

However, prior studies suggested that aripiprazole seem to be associated to a risk of inducing certain impulse control behaviors, such as uncontrollable gambling, hyper sexuality, as well as compulsive eating.

**Objectives:**

We proposed to assess the evidence for compulsive eating associated with the use of aripiprazole.

**Methods:**

We report a rare case of new onset aripiprazole induced compulsive eating behavior in a patient with bipolar disorder. Then, we conducted a literature review using“PubMed” database and keywords “Aripiprazole”, “Impulse Control Behaviors”, “Compulsive Eating”.

**Results:**

He was a 21-year-old patient, diagnosed with bipolar 1 disorder. He was prescribed Aripiprazole, after a neuroleptic malignant syndrome induced by haloperidol (which was prescribed during the first episode psychosis).

One month following the treatment initiation, the patient complained of eating excessively. He was not able to control his eating and gained 30 Kg over the period of 4 months. Metabolic assessment showed a hypercholesterolemia.

**Conclusions:**

Aripiprazole is a promising novel antipsychotic in mental diseases. However current evidence associates compulsive behaviors like eating with the use of Aripiprazole, probably due to the drug affinity to 5-HT receptors. More studies are needed to confirm this rare side effect.

**Disclosure of Interest:**

None Declared

